# Hypoproteinemia is an independent risk factor for the prognosis of severe COVID-19 patients

**DOI:** 10.3164/jcbn.20-75

**Published:** 2020-08-06

**Authors:** Liu Lin, Kaiyuan Hu, Shuijiang Cai, Xilong Deng, Xinning Shao, Ying Liang, Jigang Wang, Tianyu Zhong, Zhongwei Hu, Ming Lei

**Affiliations:** 1 Department of Nephrology, Guangzhou Medical University, No. 8, Huaying Road, Guangzhou, Guangzhou 510060, China; 2 Department of Critical Care Medicine, Guangzhou Medical University, No. 8, Huaying Road, Guangzhou, Guangzhou 510060, China; 3 Department of Artemisinin Research Center, and Institute of Chinese Materia Medical, China Academy of Chinese Medical Sciences, No. 16, Nanxiaojie, Dongzhimennei Ave, Beijing 100700, China; 4 Department of Key Laboratory of Prevention and Treatment of Cardiovascular and Cerebrovascular Diseases, Ministry of Education, Gannan Medical University, No. 1, Hexie Road, Ganzhou, Jiangxi 341000, China; 5 Department of Laboratory Medicine, First Affiliated Hospital of Gannan Medical University, No. 23, Qingnian Road, Ganzhou, Jiangxi 341000, China; 6 Gastroenterology, Guangzhou Eighth People’s Hospital, Guangzhou Medical University, No. 8, Huaying Road, Guangzhou, Guangzhou 510060, China

**Keywords:** COVID-19, albumin, prognosis, nutrition

## Abstract

Severe patients of the coronavirus disease 2019 (COVID-19) may progress rapidly to critical stage. This study aimed to identify factors useful for predicting the progress. 33 severe COVID-19 patients at the intensive care unit were included in this study. During treatment, 13 patients deteriorated and required further treatment for supporting organ function. The remaining 20 patients alleviated and were transferred to the general wards. The multivariate COX regression analyses showed that hypoproteinemia was an independent risk factor associated with deterioration of severe patients (HR, 0.763; 95% CI, 0.596 to 0.978; *p* = 0.033). The restricted cubic spline indicated that when HR = 1, the corresponding value of albumin is 29.6 g/L. We used the cutoff of 29.6 g/L to divide these patients. Kaplan–Meier curves showed that the survival rate of the high-albumin group was higher than that of the low-albumin group. Therefore, hypoalbuminemia may be an independent risk factor to evaluate poor prognosis of severely patients with COVID-19, especially when albumin levels were below 29.6 g/L.

## Introduction

The outbreak of coronavirus disease 2019 (COVID-19), caused by severe acute respiratory syndrome-associated coronavirus 2 (SARS-CoV-2), has become a pandemic. As of July 1, 2020, 10.6 million cases of COVID-19 and 0.52 million deaths had been reported. It was showed that 15.7% of COVID-19 patients became serious ill by analyzing large sample size data.^(1)^ The mortality rate of critical COVID-19 patients approached 50%.^(2)^ Clinical observation showed that among the patients who need to be transferred to the intensive care unit (ICU) for treatment, the prognosis was extremely poor if their condition progressed to the point when they needed life support, such as ventilator assisted breathing.^(3)^ In this single-center retrospective analysis, we attempted to explore the risk factors related to the prognosis of severely patients, and to provide clinicians with helpful information to identify severely patients at the early stage and treat them comprehensively and effectively.

## Subjects and Methods

### Participants

This was a retrospective single-center study. It included 33 severely patients with COVID-19 infection treated at the intensive care unit of Guangzhou Eighth People’s Hospital from January 20, 2020 to February 23, 2020. Diagnosis of COVID-19 pneumonia and clinical classification were made according to the new coronavirus pneumonia diagnosis and treatment plan (trial version 6) developed by the National Health Committee of the People’s Republic of China (http://www.nhc.gov.cn/). The clinical classifications are as follows: (1) mild, involving mild clinical symptoms and no evidence of pneumonia on imaging, (2) common, involving fever, respiratory tract issues, and other symptoms, imaging shows pneumonia, (3) severe, any of the following: a) respiratory distress, respiratory rate ≥30 beats/min; b) in the resting state, mean oxygen saturation ≤93%; c) arterial blood oxygen partial pressure/oxygen concentration ≤300 mmHg (1 mmHg = 0.133 kPa), and (4) critical, any one of the following conditions: a) respiratory failure requiring mechanical ventilation; b) shock; c) ICU admission due to combined organ failure.

### Baseline data collection

All of the patients were collected upper respiratory throat swab samples at admission which were stored in virus transport medium and transported to the Guangdong CDC for laboratory diagnosis. Epidemiological history, comorbidity, vital signs, and symptoms were recorded in detail, and various laboratory tests, including lactate dehydrogenase, creatine kinase assay, plasma albumin, blood routine, and C-reactive protein, were conducted. We also collected therapeutic measures, including oxygen therapy, use of antibiotics, fluid support (more than 1,000 ml/d), nutritional support, and application of glucocorticoids.

### Study outcome

The primary outcome was that the patients progressed to critical illness or alleviated and were transferred to general ward.

### Statistical analysis

Categorical variables were presented as number (%) and continuous variables were presented as mean (interquartile range). We compared the differences between the two groups with the *t* test, Fisher’s exact test, or Mann–Whitney *U* test. COX regression was used to select independent risk factors that affect outcomes. The corresponding albumin value when HR = 1 was found by the restricted cubic spline, and the patients were divided into the high-albumin group and the low-albumin group according to this cut-off. Kaplan–Meier curve analysis was used to determine whether there was any statistically significant difference in survival rate between the two groups. Statistical analyses were performed using SPSS, ver. 22.0, and R software (ver. R-3.5.2, www.r-project.org). *P* value <0.05 was considered statistically significant.

## Results

### Baseline data of patients

A total of 33 patients were included in this study, all of whom received intensive care in the ICU. There were 22 males (66.7%) and 11 females (33.3%), with a median age of 59 years (51.0–69.5 years). Twenty-four (72.7%) patients were suffering from basic diseases, including hypertension, diabetes, cardiovascular disease, and tumor. Additionally, the main clinical symptoms were fever, cough, shortness of breath, and diarrhea. The time from onset to treatment was 0–8 days (Table 1).

All of the patients were treated for more than 2 weeks. By February 23, 2020, 13 of the 33 patients (39.4%) had deteriorated after admission to ICU, requiring endotracheal intubation, CRRT, or ECMO treatment, and no deaths were observed. The remaining 20 patients (60.6%) became better after comprehensive treatment, such as high-concentration oxygen therapy, and were transferred to the general wards. The median time from onset to diagnosis of severe illness in 33 patients was 8 days (6.5–10.5 days) (Table 1). The median albumin value was 30.1 g/L (27.9–31.3) in all patients, 27.7 g/L (26.2–29.3) in the worsening group, and 31.0 g/L (29.9–31.6) in the improving group (Table 2).

### Associations with albumin level and prognosis in patients with COVID-19

**Fig. 1:**
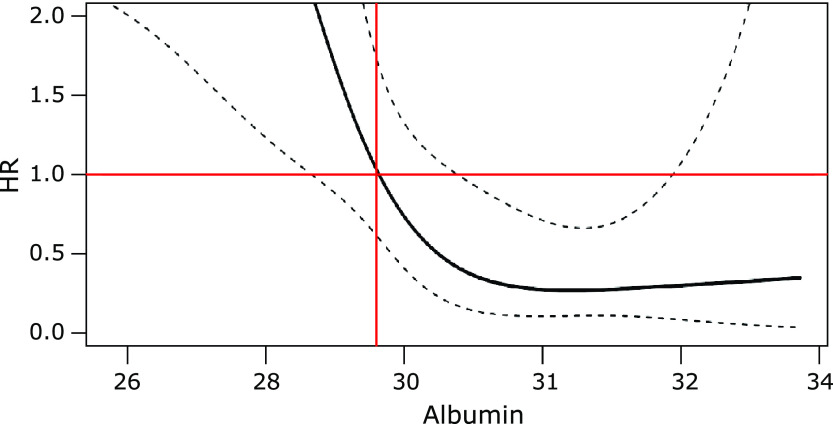
Relationship between albumin and critical ill patients. Cutoff = 29.6. HR, hazard ratio.

**Fig. 2:**
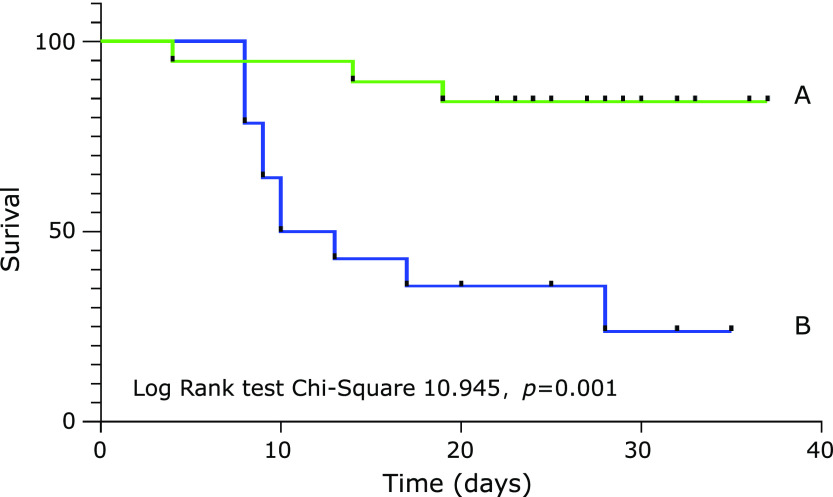
Kaplan–Meier curves. (A) The high albumin group. (B) The low albumin group.

Univariate COX regression analysis disclosed that the degree of pulmonary injury, oxygenation index, serum creatinine, urea nitrogen, leukocyte, uric acid, and albumin were associated with the prognosis of severely patients (Table 3). Multivariate COX regression analysis was performed on the significant variables in univariate COX regression analysis, suggesting that only albumin level was positively correlated to the prognosis of severe patients (HR, 0.763; 95% CI, 0.596–0.978; *p* = 0.033) (Table 4). The low level of the albumin shows the high risk of this disease. RCS analysis showed that when HR reached 1, the corresponding value of albumin was 29.6 g/L (Fig. 1). At the boundary of 29.6 g/L, the patients were divided into a high albumin group and a low albumin group. The Kaplan–Meier curve suggested that the high albumin group indicated better survival than the low albumin group (Fig. 2).

## Discussion

COVID-19 has spread throughout the world. Once the disease progresses to respiratory failure and requires ventilator assisted ventilation, the case fatality rate can reach 50%. Finding a way to reduce the progress of severely patients to critical is one of the principal goals of ICU treatment. This study showed that the median time from onset to progression to severe disease was 8 days (6.5–10.5 days). Four patients had mild symptoms at onset (presenting with low fever and mild cough), but they progressed to severe disease within 4 days. It is important to identify the risk factors for the disease progression in severely patients.

To this end, clinical manifestation, laboratory examination, and treatment data were assessed in the analysis. Univariate COX regression analysis suggested the degree of lung involvement, oxygenation index, urea nitrogen, creatinine, uric acid, albumin, and WBC were all statistically significant. The multivariable COX regression analysis to these factors indicated that albumin level was an independent risk factor for severe patients to predict the disease progression. We reviewed the clinical data of 33 patients and found that 30 cases (90.9%) had hypoalbuminemia (as indicated by local laboratory examination, under 35 g/L diagnosis hypoalbuminemia), according to the interrogation and auxiliary examination. All of 33 patients showed no evidence of liver cirrhosis, nephrotic syndrome, or chronic wasting disease. The albumin level was significantly lower in the 13 patients with advanced disease who required ventilator-assisted ventilation than in the control group. Hypoproteinemia was found to be an independent predictor of mortality.^(4–8)^ In the ICU, the incidence of hypoproteinemia was 40–50%.^(9)^ Hypoproteinemia can lead to deterioration of immune function, changes in colloid osmotic pressure, coagulation disorders, and decrease of the efficacy of certain antibiotics,^(10–13)^ thus increasing the incidence of poor prognosis in patients. These patients with COVID-19 which is a new acute respiratory infectious disease, showed obvious hypoproteinemia. Meanwhile, the incidence rate of hypoproteinemia in those patients was significantly higher than other patients in the ICU, suggesting that there is a special reason for the occurrence of hypoproteinemia in COVID-19 patients. To our knowledge, this is the first report that the hypoproteinemia is correlated to the prognosis of COVID-19 patients.

The high incidence of hypoproteinemia in COVID-19 patients implicated that most patients were in a state of protein malnutrition. The possible reasons for this are as follows: first, the digestive system, like the respiratory system, is made up of important target organs. One study found that the esophagus and ileum epithelium of the new coronavirus to be predisposing risk factors.^(14)^ Another study found the virus to be present in the feces of patients with nucleic acid.^(15)^ Some patients have diarrhea and gastrointestinal symptoms, including nausea and anorexia, which affect food intake and absorption, leading to malnutrition and hypoproteinemia. Second, hypoxia is an important initiator of pathophysiological changes in patients with COVID-19. After hypoxia, blood flow is redistributed, resulting in ischemia and hypoxia of the gastrointestinal tract earlier than in other organs. Severe COVID-19 patients have a long course of disease and are vulnerable to bacterial infection, which increases the consumption of protein and heat energy and leads to a negative nitrogen balance. Additionally, mental issues (anxiety and depression) and side effects of antiviral drugs, are prevalent in COVID-19 patients, resulting in poor appetite and reduced food intake. In summary, COVID-19 is more likely to cause protein malnutrition than other infectious diseases, and this malnutrition will further impair the immune function of patients, thus promoting the deterioration of the patients’ condition.

Currently, there are dietary recommendations for COVID-19 infection patients in China. However, in the clinical settings, especially in areas with more patients, owing to the relative lack of medical resources or the lack of participation and intervention of clinical dietitians, there are few nutrition monitoring intervention measures available for patients with severe COVID-19. Nutritional intervention is especially likely to be skipped for patients who are serious ill but still conscious and able to eat independently. In this study, patients who had undergone tracheal intubation accounted for only 54.5% of the patients who received nutritional intervention mostly consisted of small doses of intravenous amino acids and glucose. Statistical analysis did not indicate that the nutritional support based on the current regimen could speed up the recovery of COVID-19 patients. One possible explanation is that the current nutritional support was not able to be quantified and the uptake of energy and proteins in patients was not enough, which might result in the minimal effect on patient recovery.

In summary, hypoproteinemia was prevalent in patients with COVID-19 who were treated in the ICU and it was an independent risk factor for the progression to critical condition. We suggested that in the early stage of the disease, especially when the albumin level of patients was under 29.6 g/L, nutritional assessment and gastrointestinal dysfunction assessment should be conducted, and reasonable nutritional intervention measures should be taken according to the assessments. The deficiency of this study was that it included only a small number of cases and was a single-center study. At the same time, other indicators of nutritional status assessment were lacking, which makes it difficult to describe the incidence and characteristics of hypoproteinemia in severely patients with COVID-19 in a more comprehensive way. Due to time constraints, the end point of treatment was not observed. Prospective studies are still needed to establish the effect of enteral or parenteral nutritional support on the prognosis of patients infected with COVID-19.

## Conclusions

Albumin level was found to be a useful prognostic factor evaluating the prognosis for severely patients with COVID-19 pneumonia. Clinicians should be aware of the importance of the albumin level in COVID-19 patients and provide nutritional assessment and support at an early stage to reduce the incidence of critical illness.

## Figures and Tables

**Table 1:** Demographics and characteristics of patients infected with COVID-19

	All patients (*n* = 33)	Worsening group (*n* = 13)	Improving group (*n* = 20)	*p*
Age, years	58 (51.0–69.5)	65 (52.0–75.5)	57 (51.0–63.3)	0.091
Gender				0.456
Male	22 (66.7)	10 (76.9)	12 (60)	
Female	11 (33.3)	3 (23.1)	8 (40)	
Comorbid				
Diabetes mellitus	6 (18.2)	3 (23.1)	3 (15)	0.659
Hypertension	13 (39.4)	5 (38.5)	8 (40)	1
Cardiovascular disease	3 (9.1)	2 (15.4)	1 (5)	0.547
Tumor	3 (9.1)	0 (0)	3 (15)	0.261
Symptoms				
Fever	32 (97)	13 (100)	19 (95)	1
Cough	19 (57.6)	9 (69.2)	10 (50)	0.31
Shortness of breath	16 (48.5)	7 (53.8)	9 (45)	0.728
Diarrhea	4 (12.1)	3 (23.1)	1 (5)	0.276
Days from onset to treatment	3 (1.5–5)	2 (0.5–5)	3.5 (2–5.8)	0.186
Days from onset to severe	8 (6.5–10.5)	7 (6.0–9.0)	9 (7.0–11.8)	0.442
Survival time (days)	25 (11.5–28.5)	10 (8.0–15.5)	26 (25.0–32.0)	<0.001

Data are *n* (%) or median (interquartile range).

**Table 2:** Laboratory variables, CT scan and treatments of patients infected with COVID-19

	All patients (*n* = 33)	Worsening group (*n* = 13)	Improving group (*n* = 20)	*p*
Laboratory variables				
WBC (10^9^/L)	4.8 (4.2–6.3)	5.1 (4.6–7.3)	4.4 (3.8–6.1)	0.044
Neutrophil (10^9^/L)	3.6 (2.7–4.3)	3.7 (3.3–4.3)	3.4 (2.2–5.0)	0.146
Lymphocyte (10^9^/L)	1.0 (0.6–1.2)	1.0 (0.6–1.4)	1.0 (0.8–1.2)	0.668
Albumin (g/L)	30.1 (27.9–31.3)	27.7 (26.2–29.3)	31.0 (29.9–31.6)	0.001
Creatinine (µmol/L)	65.2 (45.0–90.4)	93.3 (47.2–138.4)	646 (43.3–80.6)	0.062
Urea nitrogen (mmol/L)	4.8 (3.4–6.8)	5.9 (4.0–10.5)	4.1 (3.2–6.1)	0.04
Uric acid (mmol/L)	258.4 (187.8–376.8)	333.5 (199.8–483.7)	246.0 (168.3–296.7)	0.071
Procalcitonin (ng/ml)	0.1 (0.5–0.2)	0.1 (0.1–0.2)	0.1 (0.0–0.2)	0.255
C-reactive protein (mg/L)	32.7 (19.4–54.1)	34.2 (24.0–59.0)	32.6 (14.3–57.1)	0.473
Creatine jubase (U/L)	124 (79.0–219.5)	114 (82.0–443.0)	126 (72.3–205.8)	0.326
Lactic dehydrogenase (U/L)	287 (200.0–400.0)	229 (151.0–417.0)	309 (223.8–409.0)	0.417
d-dimer (ng/ml)	1,520 (1,080–2,360)	1,440 (970–3,515)	1,590 (1,075–2,190)	0.417
CT Scan				0.054
Non apparent abnormality	3 (9.1)	0 (0)	3 (15)	
One lung is involved	3 (9.1)	3 (23.1)	0 (0)	
Both lungs are involved	27 (81.8)	10 (76.9)	17 (85)	
Treatments				
Rehydration suppor	9 (27.3)	2 (15.4)	7 (35)	0.263
Nutrition support	19 (57.6)	9 (69.2)	10 (50)	0.31
Antibiotic	32 (97)	13 (100)	19 (95)	1
Oxygenation index	157 (125.5–208.0)	127 (103.6–145.0)	184 (154.0–229.5)	<0.001

Data are *n* (%) or median (interquartile range). WBC, white blood cell.

**Table 3:** Univariate COX regression analysis

Factors	*p*
CT Scan	0.004
Oxygenation index	0.002
Creatinine	0.002
Urea nitrogen	0.001
White blood cell	0.015
Uric acid	0.009
Albumin	<0.001

**Table 4:** Multivariate COX regression analysis

Factors	HR (95% CI)	*p*
CT Scan		0.845
One lung is involved	33,988.00 (0, 2.245E+156)	0.953
Both lungs are involved	12,464.47 (0, 8.101E+155)	0.958
Oxygenation index	0.982 (0.964, 1.000)	0.051
Creatinine	0.995 (0.969, 1.022)	0.7
Urea nitrogen	1.020 (0.713, 1.459)	0.913
White blood cell	1.422 (0.921, 2.195)	0.112
Uric acid	1.004 (0.996, 1.013)	0.295
Albumin	0.763 (0.596, 0.978)	0.033

HR, hazard ratio; CI, confidence interval.
